# Exploring chamazulene as a novel therapeutic agent for breast cancer *in silico* and *in vitro*: apoptosis induction, cell cycle regulation, and antimetastatic effects

**DOI:** 10.3389/fphar.2025.1680615

**Published:** 2025-10-03

**Authors:** Liang Yao, Wanfu Wang, Bo Zhang

**Affiliations:** Department of Breast Surgery, Shanxi Province Cancer Hospital, Shanxi Hospital Affiliated to Cancer Hospital, Chinese Academy of Medical Sciences, Cancer Hospital Affiliated to Shanxi Medical University, Taiyuan, Shanxi, China

**Keywords:** breast cancer, chamazulene, apoptosis, cell cycle arrest, migration, network pharmacology, *in silico/in vitro* integration

## Abstract

**Objectives:**

This study used a combination of *in silico* and *in-vitro* methodologies to evaluate the breast cancer (BC) fighting efficacy of Chamazulene.

**Methods:**

*In silico* analyses utilized Protox-3.0 for toxicity prediction, SuperPred and GeneCards for target identification, and Jvenn for intersection. Protein–protein interactions were mapped with STRING and visualised in Cytoscape, followed by Cytohubba to pinpoint hub genes. Gene-ontology and KEGG-pathway enrichment were performed via DAVID and visualized with SRplot. Immune infiltration was assessed using TIMER, while UALCAN evaluated expression, promoter methylation, survival, and correlation. The MTT, clonogenic, EdU, Annexin-V/PI, cell cycle, wound healing, and Western-blotting were used to measure cytotoxicity and the mechanism of chamazulene in MDA-MB-231 cells.

**Results:**

*In silico* analyses indicated a safe toxicity profile and identified 53-overlapping target genes, resulting in a highly enriched PPI network. The network identified the three main hub genes: NFKB1, MAPK14, and GRB2. Enrichment analysis indicated participation in different pathways, including MAPK and HIF-1 signalling pathways. The TIMER and UALCAN investigations on BC revealed significant immune infiltration, altered gene expression, hypomethylation, and survival trends. MTT studies demonstrated a dose-dependent reduction in cancer cell viability, exceeding 50% at elevated doses. Clonogenic and EdU assays indicated reduced proliferation and DNA-synthesis, whereas apoptosis and cell cycle analyses revealed elevated cell mortality and G2/M-phase arrest. Western blotting revealed a downregulation of NFKB1, GRB2 and MAPK14, while wound healing assays suggested reduced migration.

**Conclusion:**

Chamazulene exhibits multifaceted and potent anticancer effects by specifically modifying crucial signalling-pathways and processes in aggressive BC, warranting preclinical studies to validate its therapeutic potential.

## Introduction

Breast cancer is still a worldwide health issue that touches the lives of millions of women globally (Hong and Xu, 2022). In 2025, there are expected to be 316,950 new invasive breast cancer and 59,080 non-invasive (*in situ*) breast cancer diagnoses in women within the United States alone (Breastcancer.org, 2025). Although there has been significant progress made in early detection and treatment, nearly 42,170 American women are estimated to die this year from the disease (Breastcancer.org, 2025). Current treatment modalities comprise a multi-pronged approach that includes chemotherapy, surgery, hormone therapy, radiation therapy, and targeted agents such as HER2 inhibitors and CDK4/6 inhibitors, with immunotherapy emerging as a promising addition (Trayes and Cokenakes, 2021). These interventions have significantly improved patient survival and quality of life; however, issues like therapeutic resistance, recurrence, and the insidious nature of metastasis pose persistent challenges. Though the overall incidence of breast cancer has been increasing by 1% per annum between 2012 and 2021, an even more significant increase of 1.4% per annum has been noted in women younger than 50 (Giaquinto et al., 2024). This observation highlights the need for novel therapeutic approaches and an improved understanding of the molecular pathways underlying breast cancer development.

Chamazulene, a proazulene compound that occurs mostly in the essential oil of flowers from *Matricaria recutita* L., has drawn interest due to its antioxidant and anti-inflammatory activities (Pastare et al., 2023). Recent research showed that chamazulene exhibits potential in alleviating osteoarthritic inflammation through modulating the NF-κB signaling pathway, providing the compound’s possible role in regulating inflammatory processes (Bayliak et al., 2021). In melanoma cells, chamazulene-rich essential oils from *Artemisia arborescens* (up to 63% chamazulene) induce apoptosis by modulating Bcl-2, Bax, caspase-9, PTEN, Hsp70, and SOD, disrupting cellular defense mechanisms (Russo et al., 2020). In ethanol-induced liver injury, chamazulene (25–50 mg/kg) reduces oxidative stress (lowering malondialdehyde, restoring glutathione, catalase, and SOD) and suppresses pro-inflammatory cytokines (TNF-α, IL-6), mitigating inflammation and histological damage (Wang et al., 2020). In context of the established connection between cancer development and chronic inflammation, there is a strong case to study effects of chamazulene on cancer pathways, specifically its effect on apoptosis and the cell cycle of breast cancer cells. Despite its biological activity, literature on anticancer potential of chamazulene, particularly in breast cancer, remains scarce. Our study is the first to evaluate chamazulene against breast cancer, addressing this critical research gap.

Apoptosis, essential for cellular homeostasis, removes damaged cells, but its disruption in cancer allows uncontrolled cell growth and tumor formation (Singh and Lim, 2022; Matthews et al., 2022). Metastasis, the leading cause of cancer deaths, involves cancer cells migrating through the extracellular matrix to distant tissues (Gerstberger et al., 2023). Inhibiting these pathways is a key focus of cancer research, with natural compounds like indirubin showing promise in suppressing tumor growth, regulating the cell cycle, and inducing apoptosis.

**FIGURE 1:**
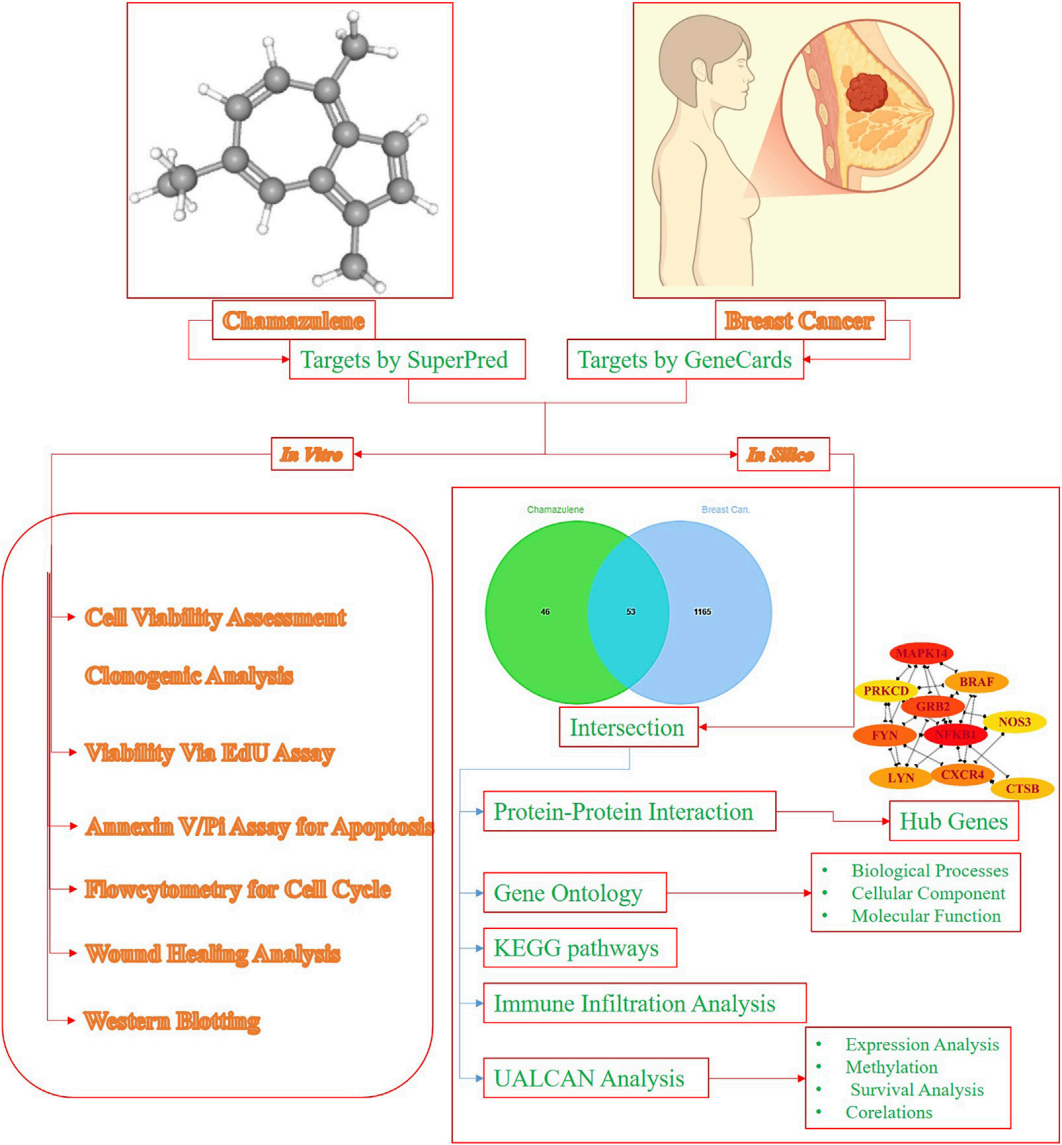
Methodology and Design of the study.

Computer-Aided Drug Design (CADD) has improved the drug-discovery process by computational techniques used to identify and optimize candidate therapeutic agents (Niazi and Mariam, 2023). For example, research has integrated network pharmacology to decipher the mechanisms of natural products such as indirubin to show their potential to suppress tumor cell growth and induce apoptosis (Jin et al., 2025). The application of CADD techniques to chamazulene may help predict its interactions with significant proteins in breast cancer development and thus speed up the drug discovery pipeline. Network pharmacology, which is an emerging field that combines systems biology and pharmacology, offers a comprehensive understanding of drug mechanisms by mapping interactions between drugs, targets, and disease pathways (Zhao et al., 2023). The approach is responsive to the fact that biological systems are complicated and diseases such as cancer have many dimensions. By developing network models, researchers can point to important nodes and pathways amenable to therapy. For instance, network pharmacology studies have been used to discover the multi-targeting mechanisms of traditional Chinese medicine compounds and reveal their therapeutic efficiency (Xin et al., 2021). This study pioneers the evaluation of breast cancer-fighting potential of chamazulene through integrated *in silico* and *in-vitro* approaches, uniquely identifying its modulation of key signaling pathways and processes like apoptosis, cell cycle arrest, and migration inhibition in aggressive MDA-MB-231 cells, providing a compelling foundation for further preclinical exploration. The detailed methodology and approach of this research have been revealed in Figure 1.

## Materials and methods

### The *in silico* analysis

#### Toxicity profiling

The initial evaluation of the safety was carried out with the Protox 3.0 online tool (“https://tox.charite.de/protox3/”) (Banerjee et al., 2018). The molecular structure of the chamazulene was uploaded to Protox 3.0, which provided predictions on toxicity endpoints, including LD_50_ values and toxicity classification. This *in silico* analysis served as a preliminary screening to assess potential adverse effects prior to further experimental investigations.

#### Target identification for chamazulene and breast cancer

Target identification for chamazulene was performed using SuperPred (“http://bioinformatics.charite.de/superpred/”), a web-based platform to predict potential molecular targets based on structural similarities and known bioactivities (Nickel et al., 2014). The targets were filtered by using a probability of ≥ 0.55%. In parallel, retrieval of breast cancer-specific gene targets from GeneCards (“https://www.genecards.org/”), a comprehensive online database that integrates gene-centric data from multiple sources (Stelzer et al., 2016). The targets were filtered by using a GIFTS score of ≥ 60. The combined data from these two platforms enabled the selection of probable targets that were relevant for further molecular investigations.

#### PPI generation

To identify shared molecular targets between chamazulene and breast cancer, the intersection of target lists was determined using Jvenn (http://jvenn.toulouse.inra.fr/), an online Venn diagram generator that allowed for a visual representation of overlapping genes (Bardou et al., 2014). The intersecting gene set was then submitted to the STRING database (“https://string-db.org/”), which generated a network of PPIs based on known and predicted associations (Szklarczyk et al., 2019). The PPI was generated with a confidence score of 0.40, and unconnected nodes were eliminated. The resulting network was imported into Cytoscape software for in-depth analysis; within Cytoscape, the Cytohubba plugin was utilized for recognition of hub targets employing the degree method, thereby highlighting key nodes that might play critical roles in disease progression and therapeutic response.

#### Gene Ontology and KEGG pathways analysis

The DAVID database (“https://davidbioinformatics.nih.gov/summary.jsp”) was used to perform functional enrichment of the intersecting genes, enabling the annotation of genes into GO categories encompassing biological processes (BP), cellular-components (CC), and molecular functions (MF). The top 15 enriched GO terms were subsequently visualized as bar plots. Additionally, the KEGG pathway information obtained from DAVID was formatted into a Sankey plot using the SRplot (“https://www.bioinformatics.com.cn/en”), providing an interactive depiction of pathway interconnections and aiding in the identification of significant metabolic and signalling cascades.

#### Immune-infiltration analysis

The TIMER online platform was used to examine hub gene expressions in the tumor microenvironment (“https://cistrome.shinyapps.io/timer/”) (Li et al., 2017). Eight different kinds of immune cells were tested for infiltration levels in connection to the expression of the target genes using this platform. The analysis offered insights into the correlation between gene expression and immune cell abundance, which is critical for understanding the immunomodulatory impact of the compound in the context of breast cancer.

#### Expression, promoter methylation, survival, and correlation analysis

The UALCAN online platform (“https://ualcan.path.uab.edu/cgi-bin/ualcan-res.pl”) enabled a comprehensive analysis of gene expression, promoter methylation, survival rates, and inter-gene correlations (Chandrashekar et al., 2017). UALCAN integrates large-scale cancer datasets to provide robust statistical analysis and visualizations, enabling the assessment of the prognostic significance and regulatory dynamics of the candidate genes in breast cancer tissues. This analysis provided critical insights into the potential clinical implications of the molecular targets identified in earlier steps.

### The *in vitro* analysis

#### Chemicals, reagents, cell lines, and antibodies

##### Chemicals and reagents

Chamazulene (7-ethyl-1,4-dimethyl-azulene, CAS 529-05-5, C_14_H_16_, 184.3 g/mol, ≥98% pure ethanol solution, catalog no. 36072) was obtained from Cayman Chemical (Ann Arbor, MI, United States). Dulbecco’s Modified Eagle Medium (DMEM, catalog no. 11965092), penicillin-streptomycin (catalog no. 15140122), and Annexin V-FITC Apoptosis Detection Kit (catalog no. 556547) were from Gibco (Thermo Fisher Scientific, Waltham, MA, United States). Fetal bovine serum (FBS, catalog no. F2442), MTT (catalog no. M2128), dimethyl sulfoxide (DMSO, catalog no. D2650), 4%-paraformaldehyde (catalog no. P6148), 0.5%-Triton X-100 (catalog no. T8787), 0.5%-crystal-violet (catalog no. C0775), and RIPA buffer (catalog no. R0278) were from Sigma-Aldrich (St. Louis, MO, United States). Methanol (catalog no. 34860) and 5% non-fat dry milk (catalog no. 1706404) were from Merck (Darmstadt, Germany). Click-iT™ EdU Imaging Kit (catalog no. C10337), Propidium Iodide (50 μg/mL, catalog no. P1304MP), and RNase A (100 μg/mL, catalog no. EN0531) were from Thermo Fisher Scientific. Protease and phosphatase inhibitor cocktail (catalog no. 04693132001) was from Roche Diagnostics (Basel, Switzerland). PVDF membranes (catalog no. IPVH00010) were from Millipore (Burlington, MA, United States). TBS-T (Tris-buffered saline with 0.1% Tween 20) was prepared using reagents from Sigma-Aldrich. Enhanced chemiluminescence (ECL, catalog no. 32106) was from Thermo Fisher Scientific.

##### Cell lines

MDA-MB-231 (triple-negative breast cancer, catalog no. HTB-26) and MCF-10A (normal epithelial, catalog no. CRL-10317) cells were procured from American Type Culture Collection (ATCC, Manassas, VA, United States). Cells were cultured in DMEM supplemented with 10% FBS and 1% penicillin-streptomycin at 37 °C in a 5% CO_2_ incubator (Thermo Fisher Scientific).

##### Antibodies

Primary antibodies against NFKB1 (catalog no. 13586, 1:1,000 dilution), MAPK14 (catalog no. 8690, 1:1,000 dilution), and GRB2 (catalog no. 3972, 1:1,000 dilution) were from Cell Signaling Technology (Danvers, MA, United States). HRP-conjugated secondary antibodies (catalog no. 1706515, 1:5,000 dilution) were from Bio-Rad Laboratories.

#### Cell culture

The MDA-MB-231 breast cancer cells (we used MDA-MB-231, a triple-negative breast cancer cell line, for its aggressive, metastatic traits, ideal for studying invasion, drug resistance, and novel therapies) and MCF-10A normal epithelial cells were procured from the American Type Culture Collection. Cells were maintained in Dulbecco’s Modified Eagle Medium (DMEM, Gibco, Thermo Fisher Scientific, Waltham, MA, United States) supplemented with 10% fetal bovine serum (FBS, Sigma-Aldrich, St. Louis, MO, United States) and 1% penicillin-streptomycin (Gibco).

#### Cytotoxicity assay

For the MTT assay, 5 × 10^3^ cells/well in 96-well plates (Corning, New York, United States) were seeded and indorsed to adhere overnight in a 5% CO_2_ incubator (37 °C; Thermo Fisher Scientific),. Cells were then exposed to the test compound at dosages of 0, 15, 30, 60, and 120 µM for 12, 24, and 48 h. Following treatment, administration of 20 μL of 5 mg/mL MTT (Sigma-Aldrich) solution was executed and incubation was extended to more 4 h (to ensure sufficient time for metabolically active cells to reduce MTT to formazan, allowing maximal color development for accurate measurement of cell viability without early saturation or underestimation) (Zulkipli et al., 2024). The insoluble formazan-crystals were dissolved in 100 μL dimethyl sulfoxide (DMSO, Sigma-Aldrich), and the absorbance was read using the Thermo Scientific Multiskan FC Microplate Photometer (Thermo Fisher Scientific). Cell viability was determined by normalizing the absorbance values to the untreated control. IC_50_ values, representing the concentration inhibiting 50% of cell viability, were determined after 48 h of treatment by fitting a dose-response curve (log [inhibitor] vs. normalized response) using GraphPad Prism software, with data expressed as mean ± SD from the triplicate experiments. The selectivity index (SI) for the Chamazulene against normal vs. breast cancer cells was determined by:

$$SI=\frac{IC50\;of\;Normal\;Cells}{IC50\;of\;Cancer\;Cells}$$

#### Clonogenic assay

To evaluate the long-term survival and proliferative capacity, cells were cultured to 6-well plates (BD Falcon, BD Biosciences, CA, United States) at 500 cells/well of concentration. Post 24-h of incubation period, administration of 0, 15, 60, and 120 µM of the compound for 48-h was performed. Post-treatment, replacing fresh medium, and allowing cells to form colonies over 10 days at 37 °C in a 5% CO_2_ incubator (Thermo Fisher Scientific). Fixing colonies with methanol (Merck, Darmstadt, Germany) was followed by stained with 0.5%-crystal-violet (Sigma-Aldrich). The colonies (≥50 cells) were manually counted under a 10x Olympus CKX41 Microscope (Olympus Corporation, Tokyo, Japan), microscope across three random fields per well in triplicate experiments to ensure reproducibility. Manual counting was executed due to the heterogeneous colony morphology and staining variability, which could lead to errors in automated systems, ensuring accurate differentiation of viable colonies from debris or overlapping clusters.

#### EdU assay

Cell-proliferation was quantified by involving Click-iT™ EdU Imaging Kit (Thermo Fisher Scientific, Waltham, MA, United States). Administration of chamazulene to MDA-MB-231 cells executed in 12-well plates (Corning) at 0, 15, 60, and 120 µM for 24 h. Incubation of cells with 10 μM EdU for 2 h at 37 °C followed treatment and subsequently cells were fixed using 4%-paraformaldehyde (Sigma-Aldrich) and permeabilized using 0.5%-Triton X-100 (Sigma-Aldrich). The EdU incorporation reaction was accomplished as per the manufacturer’s instructions, and fluorescent images were developed with the Zeiss-Axio-Observer-Inverted-Microscope (Carl Zeiss, Oberkochen, Germany).

#### Annexin-V/PI assay

Apoptotic measurements in MDA-MB-231 cells was accomplished with Annexin V-FITC and Propidium Iodide (PI). Cells seeded in 6-well plates (BD Falcon, BD Biosciences) were treated with 0, 15, 60, and 120 µM of the compound for 24 h. After treatment, cells were washed with cold phosphate-buffered saline (PBS, Gibco) and stained using the Annexin V-FITC Apoptosis Detection Kit (BD Biosciences) according to the manufacturer’s protocol. A total of 10,000 events per sample were acquired using BD Accuri C6 Flow-Cytometer (BD Biosciences), and the resultant outcomes were processed using FlowJo software (BD, Ashland, OR, United States). The gating strategy involved initial exclusion of cell debris by setting a forward scatter (FSC) versus side scatter (SSC) gate to select intact cells based on size and granularity. Doublets were excluded using FSC-area versus FSC-height plots to ensure single-cell analysis. For apoptosis assays, Annexin-V/PI staining distinguished early apoptotic (Annexin-V+/PI−), late apoptotic (Annexin-V+/PI+), and necrotic (Annexin-V−/PI+) populations.

#### Flow Cytometry

To determine the impact of chamazulene on cell cycle phases, cells were exposed to the compound at 0, 15, 60, and 120 µM for 24 h. Post-treatment, adherence of cells was maintained at −20 °C overnight in 70%-ethanol. Cells were resubjected to incubation with Propidium-Iodide (50 μg/mL) and RNase A (100 μg/mL; Thermo Fisher Scientific) for 30 min in absence of light at room temperature. The stained cells were studied using the BD Accuri C6 Flow-Cytometer (BD Biosciences) with phases distribution quantified via ModFit LT software (Verity Software House, Topsham, ME, United States). Cell cycle analysis utilized PI staining, with DNA content histograms gated to quantify G0/G1, S, and G2/M phases.

#### Cell migration assay

Evaluation of migration potency for MDA-MB-231 cells treated or untreated with chamazulene was accomplished with wound healing assay. The MDA-MB231 cells plated in 6-well plates (Corning) to 80% confluence and a linear scratch was created using a sterile 200 μL pipette tip (Eppendorf, Hamburg, Germany). After rinsing with PBS, administrating 0, 15, 60, and 120 µM of the compound in serum-free medium (Gibco). Wound condition was monitored and figured after 24-h with the Nikon Inverted Microscope (Nikon Instruments, Tokyo, Japan). The extent of migration was quantified using ImageJ software (NIH, Bethesda, MD, United States).

#### Western blotting

For protein expression studies, cells treated with 0, 15, 60, and 120 µM of the compound for 24 h were lysed in RIPA buffer (Sigma-Aldrich) containing a protease and phosphatase inhibitor cocktail (Roche Diagnostics, Basel, Switzerland). Protein concentrations were measured using the Bio-Rad Protein Assay (Bio-Rad Laboratories, Hercules, CA, United States). Equal amounts of protein (approximately 30 μg) were resolved on 10% SDS-PAGE gels using the Bio-Rad Mini-PROTEAN Tetra Cell system and subsequently transferred onto PVDF membranes (Millipore, Burlington, MA, United States) via the Trans-Blot Turbo Transfer System (Bio-Rad). Membranes were blocked with 5% non-fat dry milk (Merck) in TBS-T (Tris-buffered saline with 0.1% Tween 20, Sigma-Aldrich) for 1 h at room temperature, followed by overnight incubation at 4 °C with primary antibodies against NFKB1, MAPK14, and GRB2 (Cell Signalling Technology, Danvers, MA, United States) at optimized dilutions. After washing, membranes were incubated with HRP-conjugated secondary antibodies (Bio-Rad) for 1 h at room temperature. Protein bands were visualized using enhanced chemiluminescence (ECL, Thermo Fisher Scientific).

#### Statistical analysis

Three independent experiments, each with triplicate replicates (n = 3 per experiment), were conducted, and data were expressed as mean ± standard deviation (SD). Statistical significance was determined using GraphPad Prism Software (San Diego, CA, United States) with one-way ANOVA followed by Tukey’s post-hoc test for multiple comparisons, with p-values of *p < 0.05, **p < 0.01, and ***p < 0.001 considered significant.

## Results

### Toxicity analysis for chamazulene

The *in silico* toxicity evaluation using Protox 3.0 (Table 1) revealed a predicted LD_50_ value of 1,220 mg/kg, placing the compound in toxicity Class 4. In addition, the compound was found to be inactive for multiple toxicity endpoints, including hepatotoxicity, nephrotoxicity, respiratory, cardiac, immunologic, mutagenic, and cytotoxic responses. Notably, it demonstrated activity permeability to BBB assay while remaining inactive in both clinical and nutritional toxicity assessments. This comprehensive toxicity profile suggests that, aside from BBB penetration, the compound exhibits a relatively safe profile for further exploration.

**TABLE 1:** The *in silico* toxicity predictions for Chamazulene using the Protox 3.0 platform, including toxicity endpoints, and BBB permeability.

S.No.	Target	Prediction	Probability
1	BBB-barrier	Active	0.98
2	Cardiotoxicity	Inactive	0.82
3	Clinical toxicity	Inactive	0.82
4	Cytotoxicity	Inactive	0.75
5	Hepatotoxicity	Inactive	0.83
6	Immunotoxicity	Inactive	0.99
7	Mutagenicity	Inactive	0.71
8	Nephrotoxicity	Inactive	0.83
9	Nutritional toxicity	Inactive	0.79
10	Respiratory toxicity	Inactive	0.63

### Target identification and protein–protein interaction analysis

**FIGURE 2:**
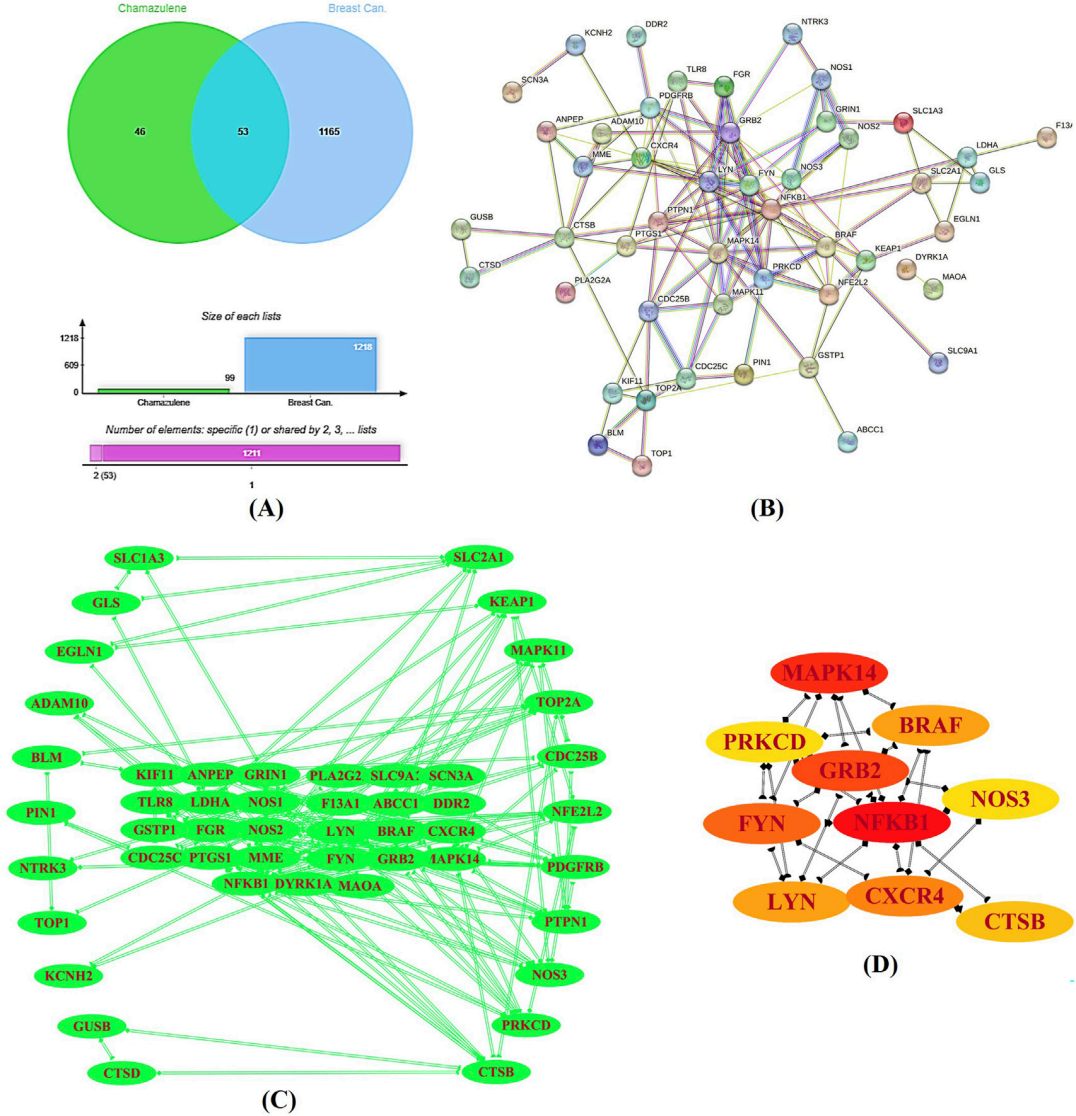
Target Identification and Protein–Protein Interaction (PPI) Analysis. **(A)** A Venn diagram generated by the Jvenn tool displays the intersection between the predicted targets for chamazulene (n = 100) and those associated with breast cancer (n = 1,218), revealing 53 common genes. **(B)** The STRING database produced a PPI network of these intersecting genes, comprising 53 nodes interconnected by 134 edges, with significant network parameters. **(C)** This network was imported into Cytoscape for enhanced visualization. **(D)** Utilizing the cytohubba plugin, key hub genes including NFKB1, MAPK14, GRB2, FYN, CXCR4, BRAF, LYN, CTSB, NOS3, and PRKCD were identified as central nodes in the network.

Initial screening identified 133 potential targets (Supplementary Table S1) for chamazulene and 18,289 for breast cancer (Supplementary Table S2). Upon applying stringent selection criteria, these numbers were refined to 100 and 1,218, respectively. The intersection of these target sets, determined using the Jvenn, yielded 53 common targets (Figure 2A). These intersecting sets were subsequently submitted to the STRING-database, which generated a PPI network comprising 53 nodes connected by 134 edges (Figure 2B). The network analysis revealed an average node degree and local clustering coefficient of 5.06 and 0.5, far exceeding the expected 58 edges. The enrichment p-value was calculated to be less than 1.0e-16, signifying a highly significant interaction network. Visualization of the network in Cytoscape (Figure 2C), aided by the Cytohubba plugin, enabled the documentation of key hub genes, namely, NFKB1, MAPK14, GRB2, FYN, CXCR4, BRAF, LYN, CTSB, NOS3, and PRKCD (Figure 2D). These hub genes likely represent critical nodes in the modulation of cellular processes relevant to breast cancer pathology.

### Functional enrichment and pathway analysis

**FIGURE 3:**
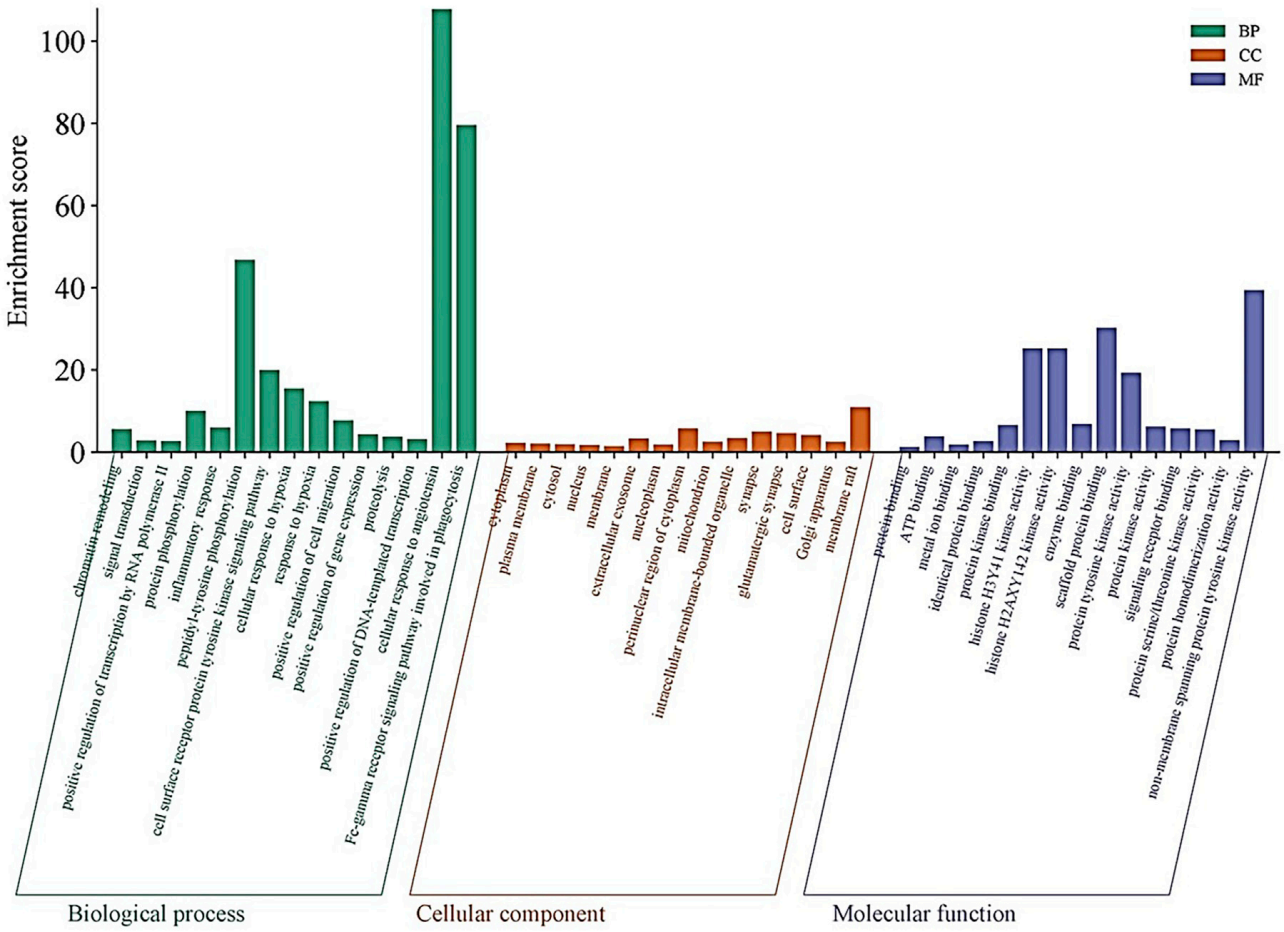
Gene Ontology (GO) Enrichment Analysis: Bar plots illustrate the enriched GO terms for the 53 intersecting genes across three categories: biological processes (BP), cellular components (CC), and molecular functions (MF). Highly enriched terms include chromatin remodeling, signal transduction, protein phosphorylation, and inflammatory response in BP; key subcellular localizations such as the cytoplasm, plasma membrane, and extracellular exosome in CC; and ATP binding, metal ion binding, and non-membrane spanning protein tyrosine kinase activity in MF.

The DAVID database aided further evaluation of the 53 intersecting genes for their functional roles through GO and KEGG pathway enrichment. In the BP category, several terms were highly enriched, including chromatin remodelling (enrichment score: 5.59), signal transduction (2.76), positive regulation of transcription by RNA polymerase II (2.65), protein-phosphorylation (10.04), and inflammatory response (6.05). Particularly striking was the enrichment for peptidyl-tyrosine phosphorylation (46.77) and the cell surface receptor protein tyrosine kinase signalling pathway (19.98) (Figure 3). Within the CC category, the analysis highlighted various subcellular localizations such as the cytoplasm (2.19), plasma membrane (1.97), cytosol (1.89), nucleus (1.72), and the extracellular exosome (3.30). Additionally, components like the membrane raft (10.93) and perinuclear region of the cytoplasm (5.67) were enriched (Figure 3). In the MF category, functions such as ATP binding (3.76), metal ion binding (1.78), protein kinase binding (6.58), and non-membrane-spanning-protein-tyrosine-kinase-activity (39.39) were significantly represented. Other MF terms, including histone H3Y41 and H2AXY142 kinase activities (both 25.21) and scaffold protein binding (30.2) further emphasize the complex regulatory capabilities of these genes (Figure 3).

**FIGURE 4:**
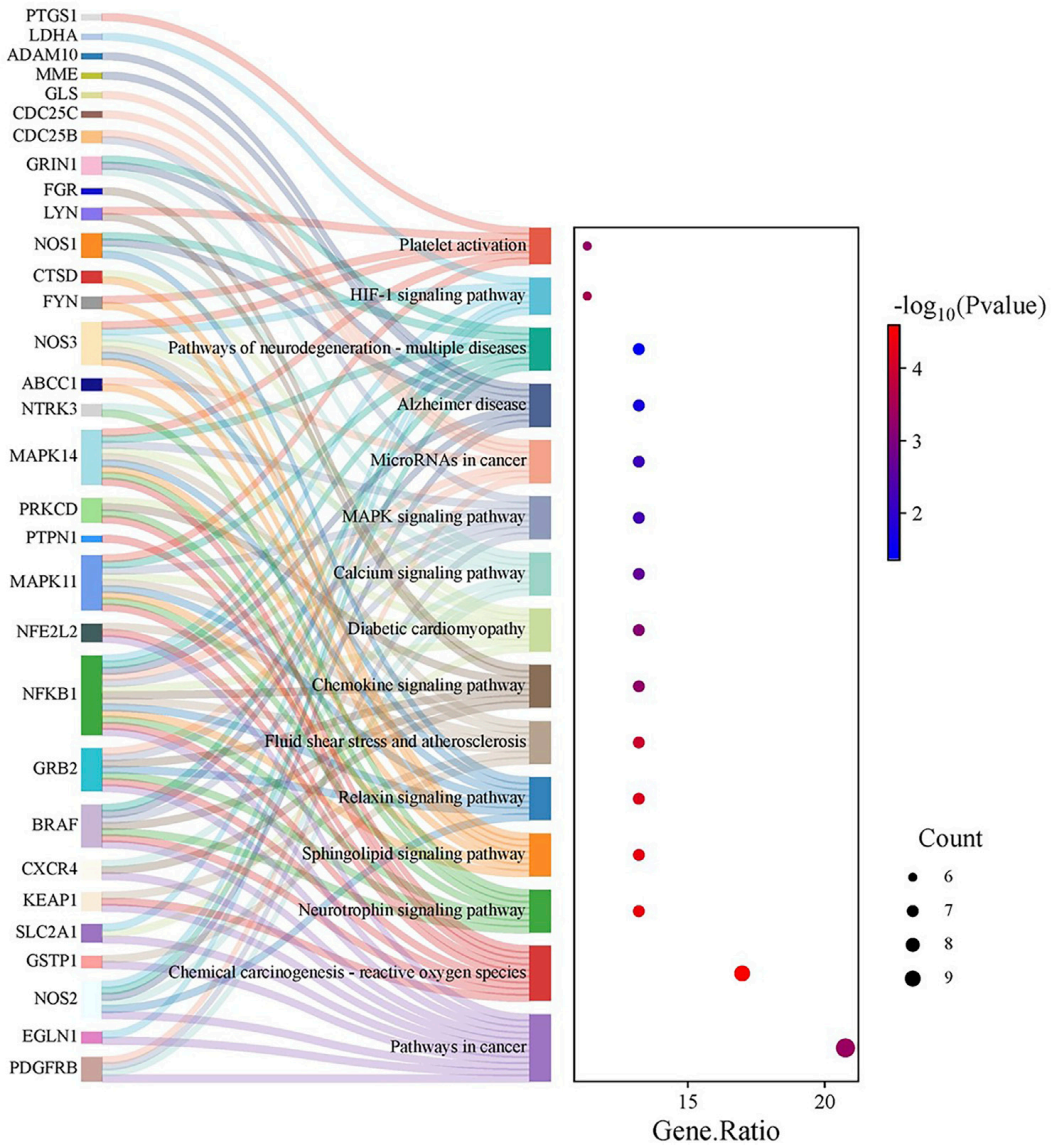
KEGG Pathway Enrichment Analysis: A Sankey plot (or equivalent visualization) delineates the enriched KEGG pathways linked to the intersecting genes. Notable pathways include those related to cancer, chemical carcinogenesis via reactive oxygen species, neurotrophin, sphingolipid, relaxin, chemokine, and MAPK signalling, among others, highlighting the compound’s potential impact on diverse oncogenic processes.

KEGG pathway exploration demonstrated the implication of intersecting genes in multiple signalling pathways and metabolic networks relevant to cancer. The enriched pathways include those directly allied to cancer, chemical-carcinogenesis via ROS, neurotrophin, sphingolipid, and relaxin signalling pathways, as well as fluid shear stress and atherosclerosis. Other significant pathways were the chemokine signalling pathway, diabetic cardiomyopathy, calcium signalling, MAPK signalling, microRNAs in cancer, Alzheimer’s disease, neurodegeneration (multiple diseases), HIF-1 signalling, and platelet activation (Figure 4).

### Immune infiltration analysis

**FIGURE 5:**
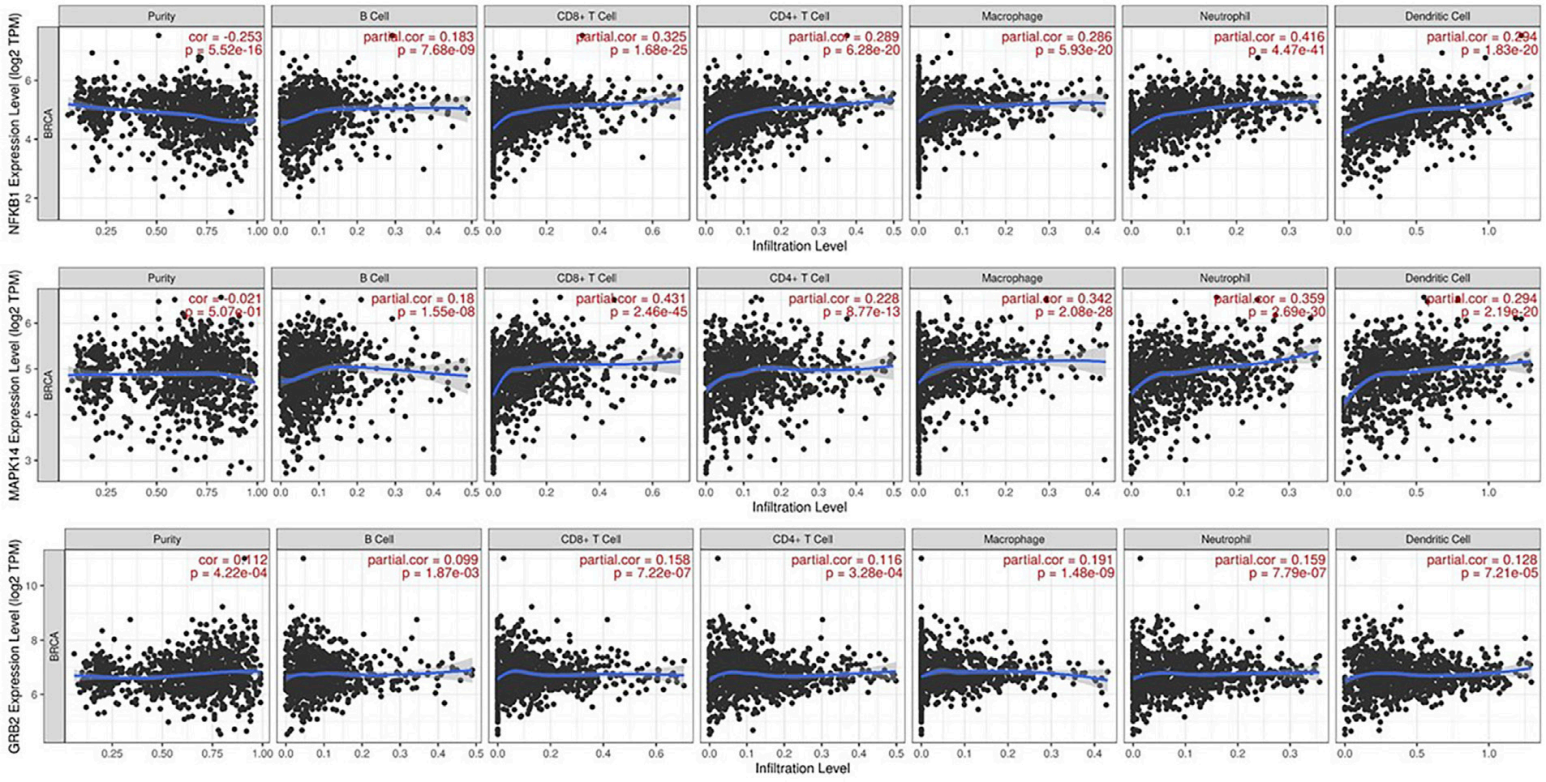
Immune Infiltration Analysis via TIMER: Scatter plots from the TIMER platform demonstrate the correlations between the expression levels of NFKB1, MAPK14, and GRB2 and the degree of infiltration by various immune cell subtypes (e.g., B cells, CD4^+^ T cells, macrophages) in breast cancer tissues. Each panel includes the partial correlation coefficient and p-value, indicating significant positive associations.

The TIMER online platform found a correlation between NFKB1, MAPK14, and GRB2 gene expression and infiltration in TME in breast cancer (Figure 5). In each image, the horizontal axis shows immune cell subtype infiltration (e.g., B cells, CD4^+^ T cells, macrophages), while the vertical axis shows the gene of interest normalized transcript level (log2 TPM). Each graph shows the partial correlation coefficient (partial r) and p-value in the top right corner. The three genes have a positive connection with numerous immune cell types, indicating that greater NFKB1, MAPK14, or GRB2 expression may boost tumor microenvironment immune cell infiltration. NFKB1 and MAPK14 have modest relationships with macrophage and dendritic cell infiltration, suggesting they may modulate inflammatory or antigen-presenting pathways. GRB2 has a favourable connection with B cells, neutrophils, and other leukocyte subsets, suggesting it may be involved in immune surveillance signalling cascades. The substantial associations (p < 0.05) suggest these genes may be immunologically important indicators in breast cancer.

### Expression, promoter methylation, survival, and correlation

**FIGURE 6:**
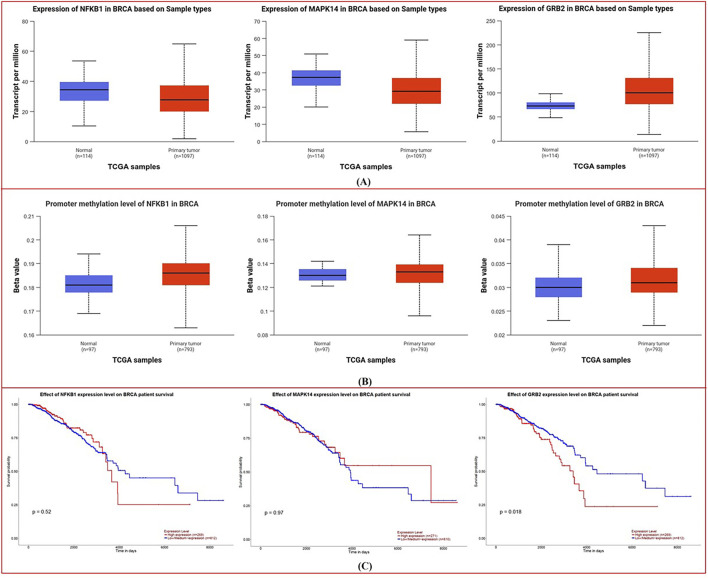
Expression, Promoter Methylation, and Survival Analysis from UALCAN: **(A)** Boxplots compare the expression levels of NFKB1, MAPK14, and GRB2 in normal versus tumor tissues, showing elevated transcript levels in breast cancer. **(B)** Corresponding boxplots of promoter methylation reveal lower methylation in tumor samples. **(C)** Kaplan–Meier survival curves illustrate distinct survival trends based on differential gene expression, suggesting prognostic implications.

UALCAN platform-generated boxplots comparing normal and tumor tissue expression (A), promoter methylation levels (B), and Kaplan–Meier survival curves (C) for NFKB1, MAPK14, and GRB2 are shown in Figure 6.

Expression Analysis: Boxplots (Figure 6A) show considerable changes in transcript abundance amid normal vs. tumor samples. In breast cancer tissues, NFKB1, MAPK14, and GRB2 are more highly expressed than in normal tissues, although the differences vary. Significant differences imply overexpression of these genes in malignant transformation, according to statistical analysis.

Methylation status in normal and malignant tissues is compared in the boxplots (Figure 6B). Tumor samples had reduced methylation for each gene, although the amount varies. Breast cancer cells may exhibit elevated NFKB1, MAPK14, and GRB2 expression due to a reduction in promoter methylation.

Kaplan-Meier plots (Figure 6C) show survival variations depending on gene expression levels. GRB2’s survival curves vary more than NFKB1 and MAPK14’s (p-values are lower). This implies that higher GRB2 expression may be linked to a survival trend, suggesting its prognostic relevance in breast cancer.

**FIGURE 7:**
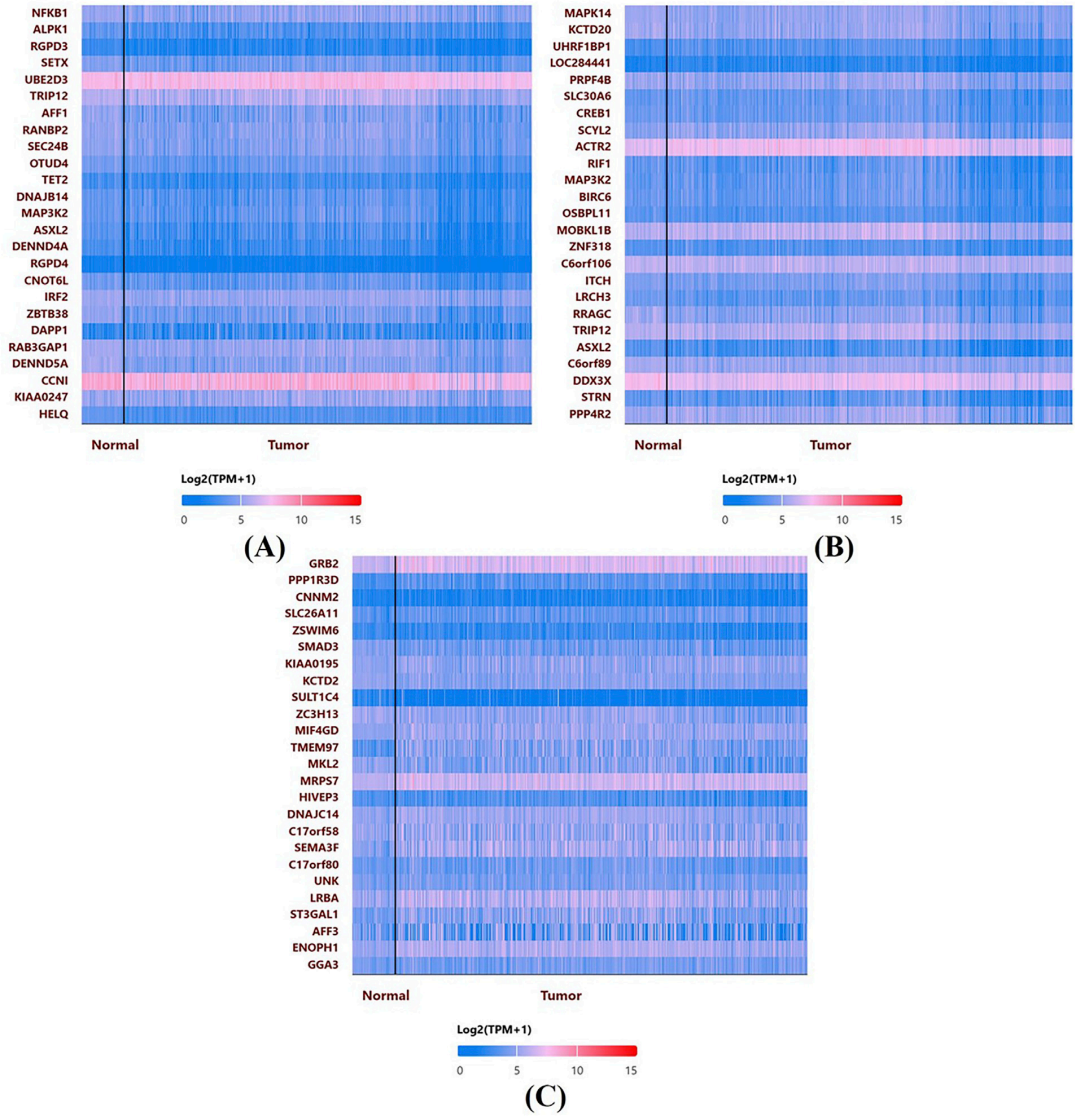
Co-expression Heatmaps from UALCAN: Heatmaps display the expression patterns of genes co-expressed with NFKB1 **(A)**, MAPK14 **(B)**, and GRB2 **(C)** in normal and malignant breast tissues. The color scale (log2 TPM + 1) indicates higher expression in red and lower in blue, highlighting distinct clustering patterns and potential co-regulatory networks.

In Figure 7, UALCAN heatmaps show the expression patterns of genes co-expressed or possibly linked with NFKB1 (A), MAPK14 (B), and GRB2 (C) in normal and malignant breast tissues. Red signifies high expression and blue suggests low expression on the color scale (log_2_ TPM + 1). Multiple genes express differently in normal and malignant samples in each heatmap. Many genes associated with NFKB1, MAPK14, or GRB2 are elevated in tumor tissues, suggesting co-regulatory networks or signalling pathways. These hub genes often cluster with genes involved in cell cycle control, kinase activity, and inflammation. The clustering patterns imply that NFKB1, MAPK14, and GRB2 may work with other oncogenes or tumor suppressors to shape breast cancer biology.

### Induction of cytotoxicity

**FIGURE 8:**
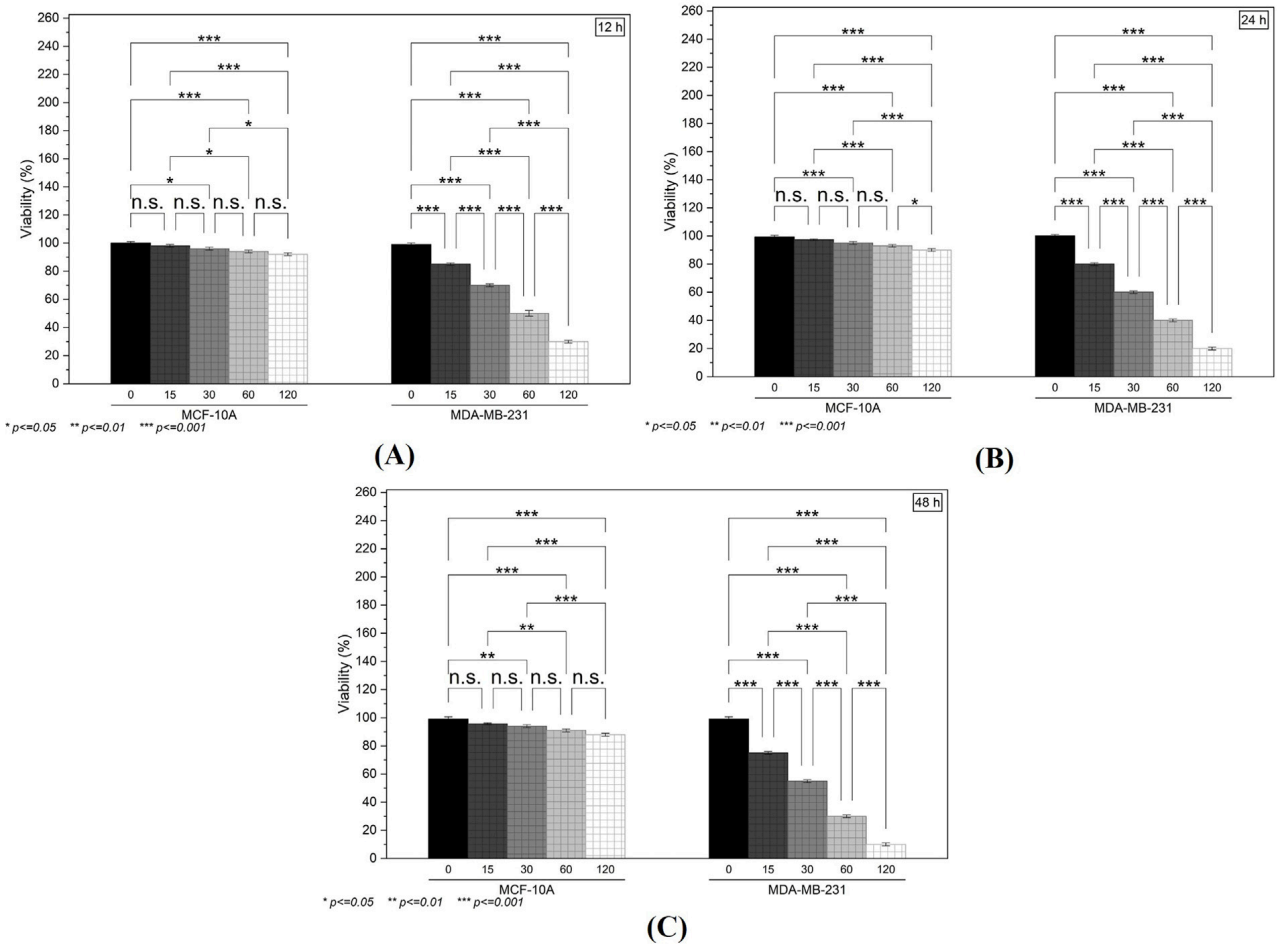
The *in vitro* Cytotoxicity and Clonogenic Assays: **(A–C)** MTT assay results at 12, 24, and 48 h illustrate a concentration-dependent reduction in the viability of MDA-MB-231 cells, with minimal effects on MCF-10A cells.

Treatment with the test compound produced a clear, concentration-dependent reduction in the viability of MDA-MB-231 cells over the 12- (Figure 8A), 24- (Figure 8B), and 48-h (Figure 8C) intervals. Notably, at higher doses (60 and 120 µM), cell viability decreased by more than 50% compared to the untreated group. In contrast, MCF-10A cells showed relatively modest changes in metabolic activity, with only minimal reductions observed at the highest concentration. The IC_50_ values for chamazulene were 71 µM against MCF-10A normal breast cells and 41 µM against MDA-MB-231 breast cancer cells, yielding a selectivity index (SI) of 1.7 (IC_50_ normal/IC_50_ cancer). This SI indicates desirable selective toxicity, preferentially targeting cancer cells while sparing normal cells. These findings suggest that the compound selectively impairs the metabolic function of malignant cells while exerting limited cytotoxicity on normal epithelial cells.

### Suppression of clonogenic potency

**FIGURE 9:**
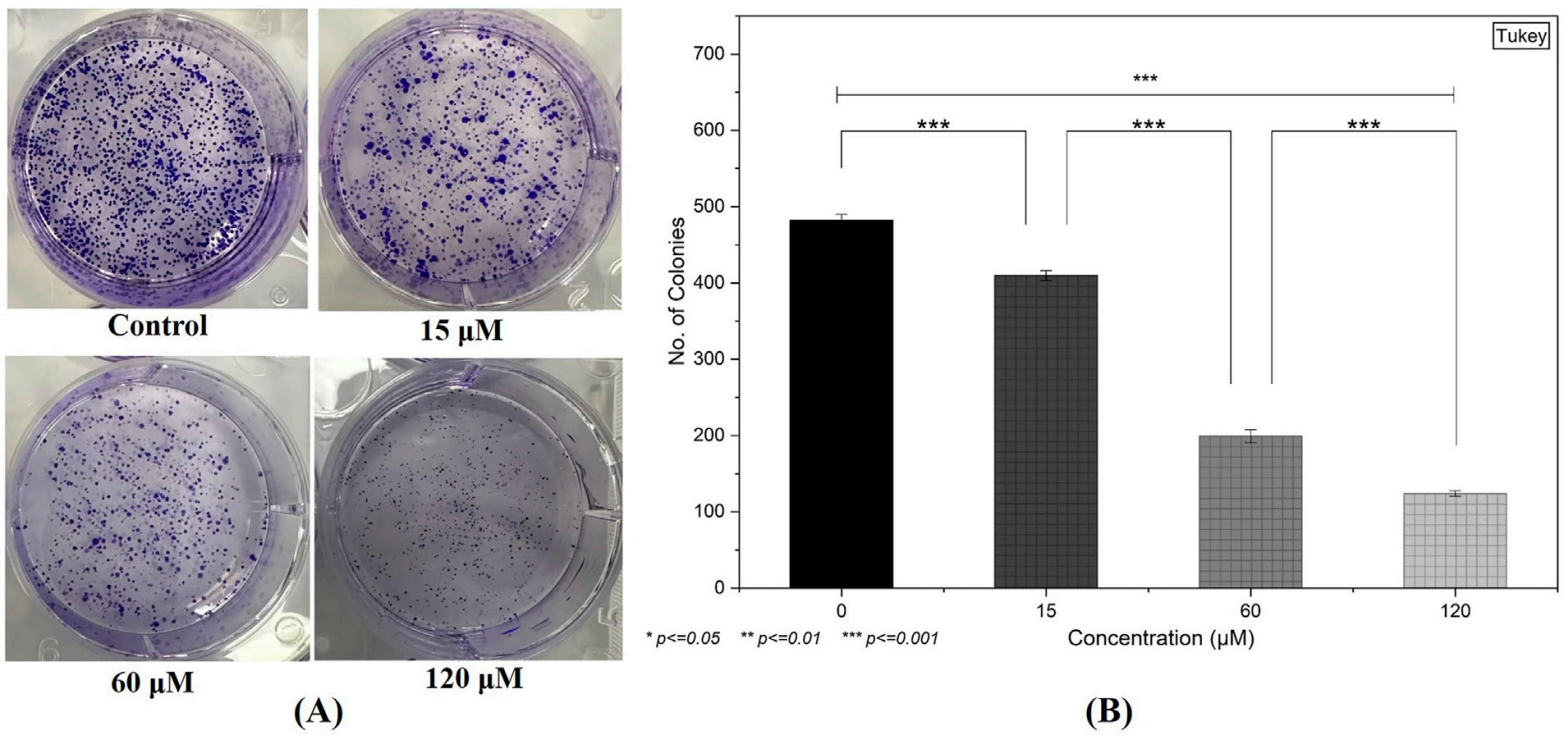
Inhibition of Clonogenic Potency: **(A)** A clonogenic assay demonstrates a substantial decline in colony formation in MDA-MB-231 cells following 48-h treatment, particularly at higher compound concentrations. **(B)** Quantification of the colonies by selecting three random fields from each treatment group. (Individual experiments were given triplicate replicas; n = 3; *p < 0.05, **p < 0.01 and *p < 0.001)).

Following a 48-h exposure to 0, 15, 60, and 120 µM of the compound, the ability of MDA-MB-231 cells to form colonies was notably hindered (Figure 9A). Untreated controls exhibited a high density of well-defined colonies, whereas cells treated with 15 µM showed a moderate decline in colony count. This decrease became more pronounced at 60 and 120 μM, where only a few discrete colonies were visible (Figure 9B). These results indicate a substantial inhibitory effect on the long-term proliferative capacity of the breast cancer cells, aligning with the cytotoxicity data.

### Inhibition of DNA synthesis

**FIGURE 10:**
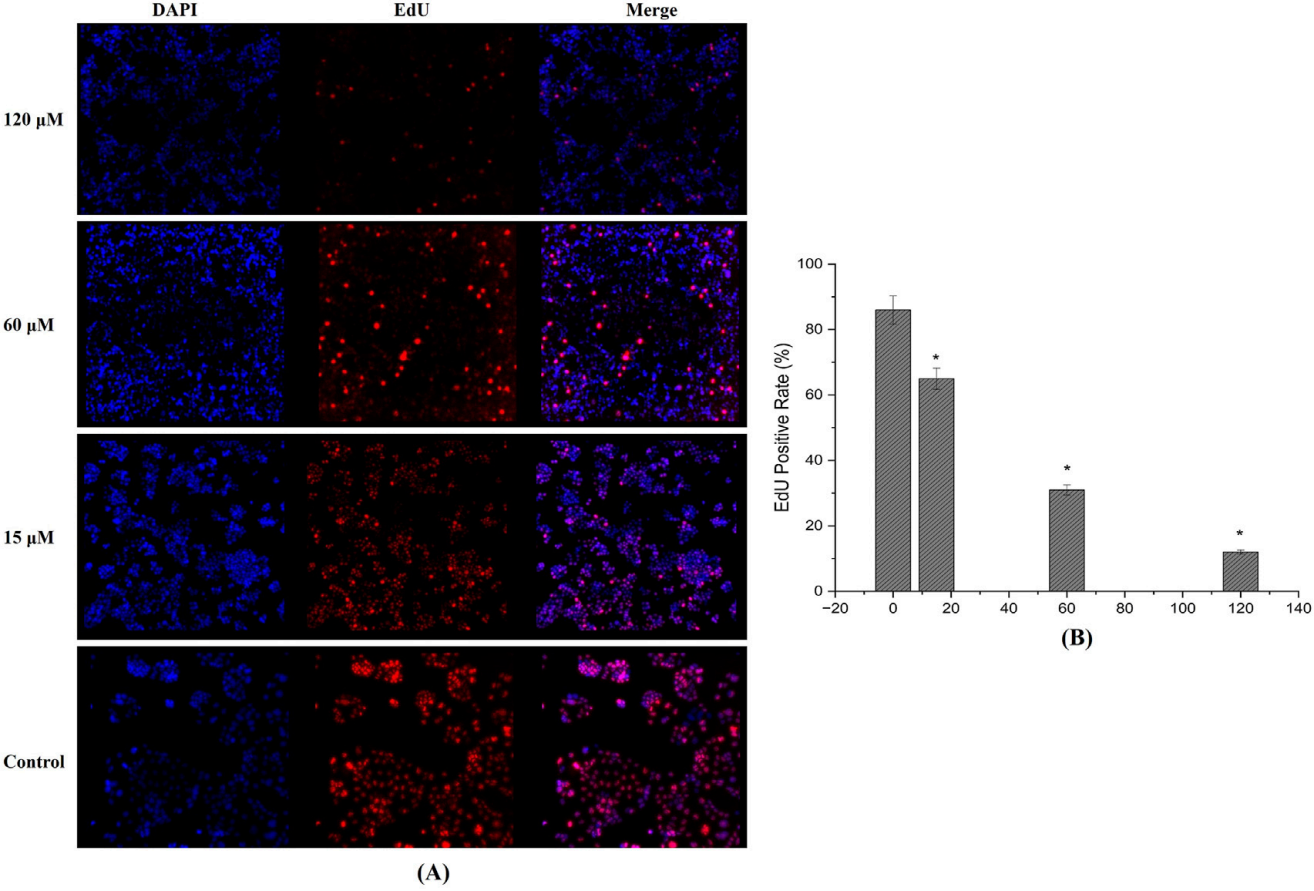
EdU Incorporation Assay: **(A)** Fluorescence images reveal a dose-dependent decrease in EdU-positive MDA-MB-231 cells after 24-h treatment, indicating significant inhibition of DNA synthesis and cell proliferation. **(B)** EdU positive cells quantified using fluorescence intensity showing reduction and inhibition DNA synthesis. (Individual experiments were given triplicate replicas; n = 3; *p < 0.05).

EdU incorporation analysis further confirmed the anti-proliferative properties of the test compound. In MDA-MB-231 cells treated for 24 h, a dose-dependent drop in EdU-positive cells was observed, implying a significant reduction in DNA synthesis (Figure 10A). At 15 μM, a moderate decrease in fluorescent signal was noted, whereas concentrations of 60 µM and above displayed markedly lower levels of newly synthesized DNA (Figure 10B). This reduction in EdU incorporation corroborates the compound’s potential to impede cell proliferation.

### Blocking cell cycle

**FIGURE 11:**
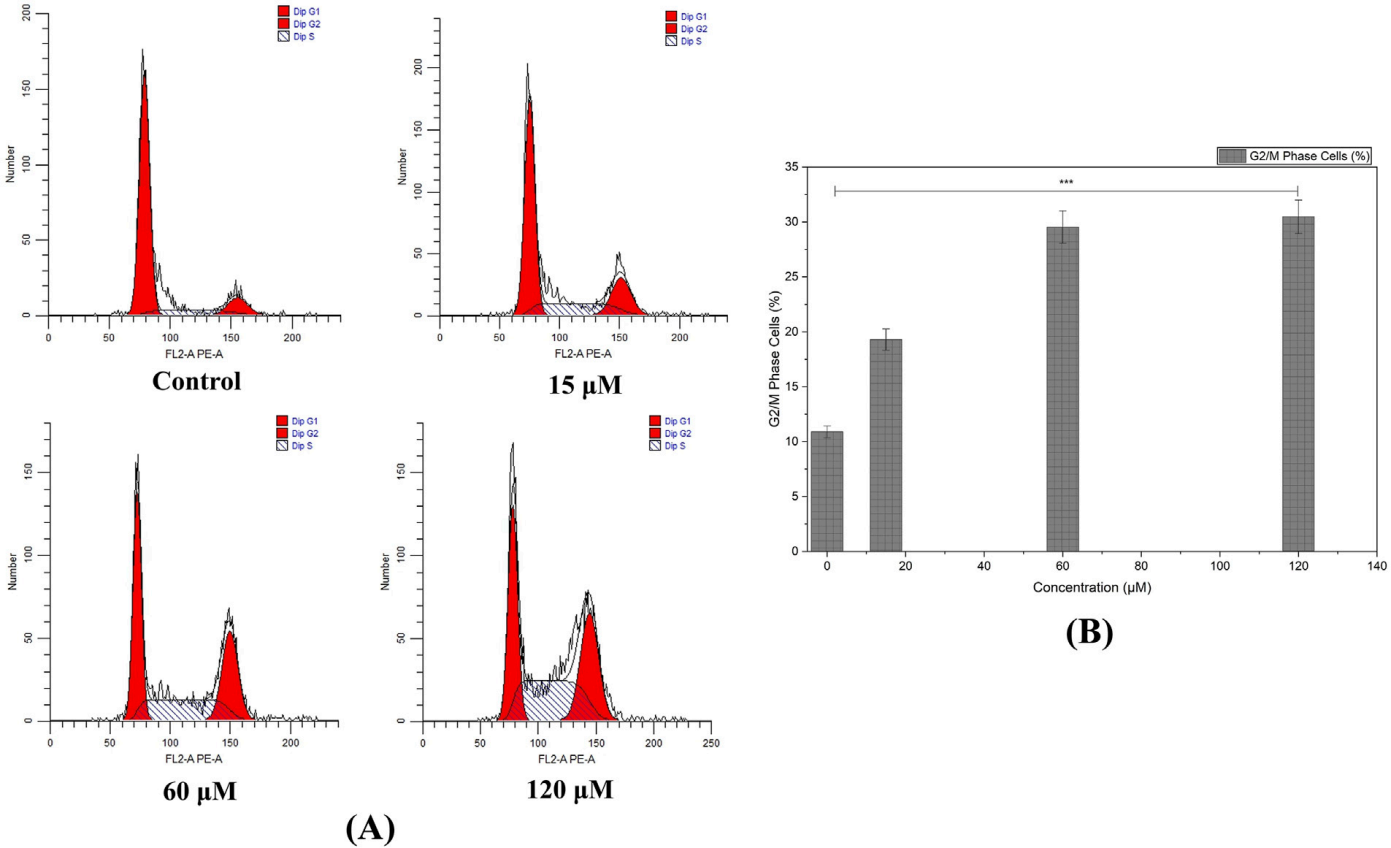
Cell Cycle Analysis by Flow Cytometry: **(A)** Histograms illustrate the accumulation of MDA-MB-231 cells in the G2/M phase following treatment with higher concentrations of the compound, indicating cell cycle arrest and a potential checkpoint blockade. **(B)** Augmentation in G_2_/M phase cells with increased treatment concentration of Chamazulene. (Individual experiments were given triplicate replicas; n = 3; *p < 0.05, **p < 0.01 and *p < 0.001)).

Flow cytometric examination revealed that rising concentrations of the compound led to cell-cycle perturbations in MDA-MB-231 cells. We identified marked accumulation in G_2_/M phase as recorded at 60 and 120 μM, suggesting that the compound triggers a checkpoint blockade or delays mitotic progression (Figures 11A,B).

### Triggering of apoptosis

**FIGURE 12:**
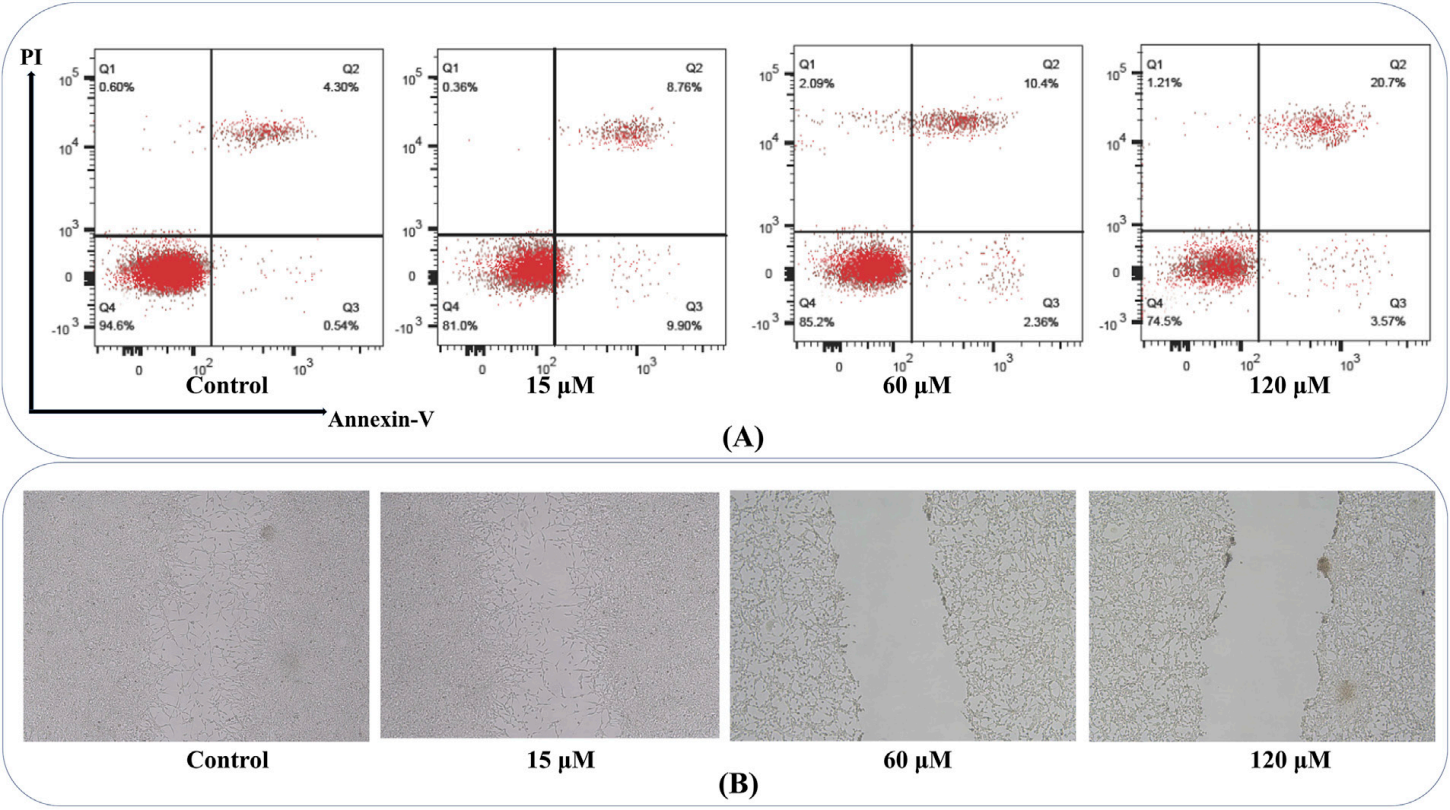
Annexin V-FITC/PI Apoptosis and Wound Healing Assay: **(A)** Flow cytometry plots show an increased percentage of early and late apoptotic MDA-MB-231 cells with escalating compound doses, confirming the induction of apoptosis in a concentration-dependent manner. Q1 represents necrotic cells (Annexin V–/PI+); Q2 shows late apoptotic or secondary necrotic cells (Annexin V+/PI+); Q3 includes healthy, viable cells (Annexin V–/PI–); Q4 identifies early apoptotic cells (Annexin V+/PI–). **(B)** Representative images of scratch assays demonstrate that MDA-MB-231 cells treated with higher compound concentrations exhibit delayed wound closure compared to controls, reflecting impaired migratory capacity and potential anti-metastatic effects.

Apoptosis evaluation revealed that the percentage of apoptotic cells (early and late) rose in a concentration-dependent pattern in MDA-MB-231 cultures (Figure 12A). At 15 μM, an appreciable shift toward early apoptotic cells was detected, while 60 μM and 120 µM treatments significantly increased both early and late apoptosis.

### Triggering wound closure

Wound healing measurements demonstrated a significant reduction in migratory capacity among MDA-MB-231 cells administrated higher chamazulene concentrations. Scratches in control wells showed substantial closure within 24 h, while those exposed to 60 μM and 120 µM retained a notably broader gap, indicative of impaired cell migration (Figure 12B). These findings point to the compound’s potential in limiting metastatic behavior by suppressing migratory processes in aggressive breast cancer cells.

### Expression of hub genes

**FIGURE 13:**
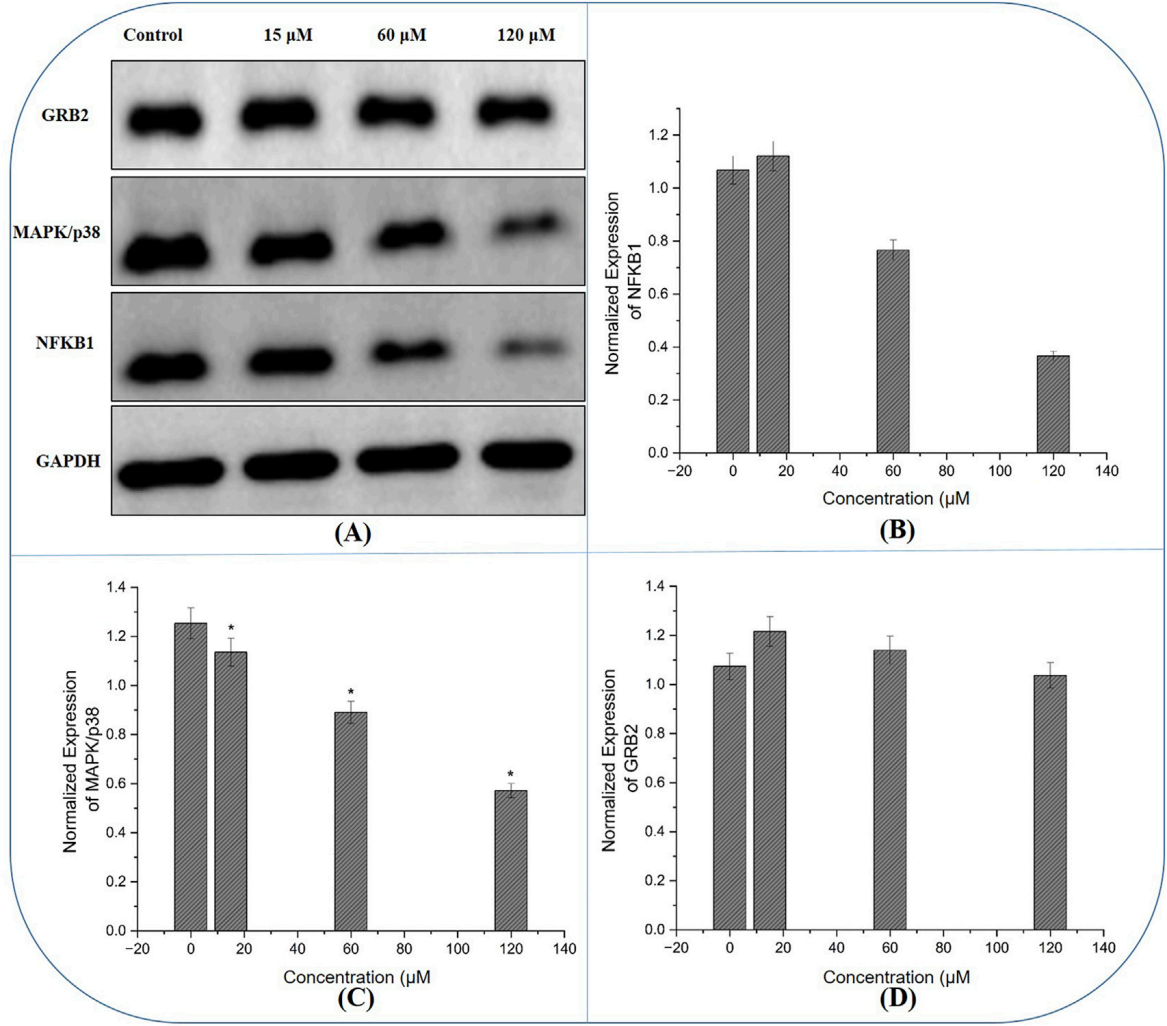
Western blot Analysis of Hub Gene Expression: **(A)** Immunoblot images reveal that treatment with the compound results in reduced protein levels of NFKB1 and GRB2, alongside the expression of MAPK14 in MDA-MB-231 cells, with the most pronounced changes observed at higher concentrations, corroborating the compound’s molecular impact on key oncogenic signalling pathways. **(B–D)** The normalized expression of NFKB1, GRB2 and MAPK14 generated using fluorescence intensity with ImageJ software.

Western blot results provided insight into the molecular events underpinning the compound’s anticancer activity. Compared to untreated controls, cells exposed to 60 μM and 120 µM exhibited reduced protein levels of NFKB1, GRB2 and MAPK14 (Figures 13A–D). Densitometric quantification showed that these regulations were most pronounced at the highest concentration, aligning with the functional assays.

## Discussion

Our research combines molecular insights with both computational and experimental data to investigate the anticancer efficacy of a chamazulene in breast cancer. The *in silico* research, including target identification and PPI network design, revealed 53 overlapping genes between chamazulene targets and breast cancer-associated genes, with NFKB1, MAPK14, and GRB2 identified as pivotal hub genes. Functional enrichment analysis corroborated that these genes are pivotal to pathways associated with protein phosphorylation, inflammatory responses, and cell migration, which are essential in cancer development.

NFKB1, MAPK14, and GRB2 are essential regulators in the oncogenic environment, each playing a distinct role in cancer development across several malignancies. NFKB1, an essential member of the NF-κB transcription factor family, regulates genes associated with inflammation, cellular survival, and proliferation (Zinatizadeh et al., 2021). Its continuous activation has been associated with carcinogenesis, resistance to apoptosis, and metastasis in several malignancies (Schlein and Thamm, 2022). Aberrant NF-κB signalling modulates the pro-inflammatory milieu that triggers cell survival, augments angiogenesis, and facilitates invasion, positioning NFKB1 as a pivotal target for anticancer therapy (Chrysanthakopoulos and Vryzaki, 2023).

MAPK14, or p38α, is a stress-activated protein kinase that has a dual function in cancer. MAPK14 facilitates responses to environmental stress and inflammatory signals, playing a role in the activation of cell cycle checkpoints and the initiation of apoptosis (García-Hernández et al., 2021). Conversely, its function may vary with environment, since p38 MAPK activation may also aid cell survival under some circumstances (Kudaravalli et al., 2022). In malignancies, this equilibrium is often disturbed, resulting in either unregulated growth or resistance to apoptosis. Consequently, regulating MAPK14 activity is a prospective therapeutic strategy to reestablish appropriate cell cycle regulation and activate apoptotic pathways in malignant cells (Xu et al., 2021).

GRB2 functions as a vital adapter protein that transmits signals from receptor tyrosine kinases to downstream effectors, including the Ras/MAPK pathway (Wang et al., 2024). GRB2’s role in connecting extracellular signals to intracellular responses positions it centrally in cellular processes including growth, differentiation, and migration (Malagrinò et al., 2024). In several malignancies, increased GRB2 levels have been linked to augmented oncogenic signalling, which facilitates the unchecked proliferation and metastasis (Ye et al., 2024). Disruption of GRB2-mediated pathways might impede the signal transduction necessary for tumor proliferation, making it a compelling target in cancer therapeutic efforts.

NFKB1, MAPK14, and GRB2, identified as top hub genes via network pharmacology, play crucial roles in breast cancer pathways. NFKB1 activates NF-κB signaling, promoting inflammation, survival, and metastasis. MAPK14 modulates p38 stress responses, enhancing tumor growth and invasion. GRB2 links RTKs to Ras/MAPK, driving proliferation. These *in silico* insights augment our results, serving as basis for *in vitro* analysis. (Zinatizadeh et al., 2021; Schlein and Thamm, 2022; Chrysanthakopoulos and Vryzaki, 2023; García-Hernández et al., 2021; Kudaravalli et al., 2022; Xu et al., 2021; Wang et al., 2024; Malagrinò et al., 2024; Ye et al., 2024).

*In vitro* experiments corroborated these results. Cytotoxicity studies revealed a concentration-dependent decline in the viability of MDA-MB-231 cells, but normal MCF-10A cells had no impact. Clonogenic and EdU tests demonstrated substantial suppression of proliferation and DNA synthesis in malignant cells. Apoptosis tests and cell cycle analysis demonstrated amplified cell death and G_2_/M arrest after treatment, indicating effective interruption of survival and proliferative signals.

The Western blot analysis confirmed our network findings: the chamazulene treatment resulted in decreased protein levels of NFKB1 and GRB2, presumably contributing to the inhibition of survival and proliferative signals in breast cancer cells. Conversely, MAPK14 levels were downregulated, indicating that the suppression of this stress-response kinase may trigger cell cycle arrest and apoptosis. These findings together suggest that altering these three critical regulators might significantly diminish cancer cell viability and metastatic capability.

The anticancer effects observed for chamazulene are consistent with those established for natural products like curcumin and resveratrol. Curcumin targets several cellular pathways, suppressing cell proliferation, migration, and stemness; promoting apoptosis, autophagy, and ferroptosis; and dampening inflammation and angiogenesis (Li et al., 2020; Tong et al., 2020; Tian et al., 2021). Resveratrol efficiently induces programmed cell death through modulation of apoptosis-, autophagy-, and necroptosis-related pathways, including PI3K/AKT, MAPK, and NF-κB, regulating pro- and anti-apoptotic proteins and inhibiting cell survival, angiogenesis, and inflammation (Wu et al., 2023; Jang et al., 2022; Chhabra et al., 2021). Similarly, chamazulene demonstrates inhibition of cell cycle progression and cell proliferation, reduced migration, and induction of apoptosis, with marked involvement of MAPK, NF-κB, and GRB2 pathways. These mechanistic similarities position chamazulene alongside curcumin and resveratrol as promising natural product-based therapies, as it shares overlapping molecular targets and biological activities in anticancer mechanisms. This highlights the therapeutic potential of chamazulene within the broader spectrum of well-characterized phytochemicals for cancer management.

This study integrates *in silico* network pharmacology with comprehensive *in vitro* assays (MTT, clonogenic, EdU, apoptosis, cell cycle, wound healing, Western blot), demonstrating chamazulene’s novel anticancer potential through apoptosis induction, cell cycle arrest, and reduced migration. However, limitations include reliance on a single cancer cell line, lack of *in vivo* validation, and limited normal cell testing. These gaps offer exciting opportunities for future studies to explore diverse cell lines, *in vivo* models, and broader safety profiles, enhancing translational potential.

## Conclusion

Our research, integrating *in silico* predictions with rigorous *in vitro* studies, reveals a promising anticancer profile for the chamazulene against breast cancer. The toxicity evaluation and target identification identified key molecular components involved in tumor growth, while network analysis highlighted its impact on cell proliferation, inflammation, and migratory pathways. TIMER analysis indicated substantial immune cell infiltration linked to key hub genes, suggesting a possible function in regulating the tumor microenvironment. UALCAN data further substantiated these results by demonstrating differential gene expression, modified promoter methylation, and significant survival trends in breast cancer patients; therefore, it affirms the therapeutic significance of the identified targets. The *in vitro* experiments demonstrated that the chamazulene preferentially reduces the viability and clonogenic capacity of cancer cells. It substantially inhibited DNA synthesis, triggered apoptosis, disturbed cell cycle progression, and reduced cellular migration in wound-healing experiment. The Western blot analysis confirmed molecular validity by showing the regulation of key signalling proteins associated with carcinogenic pathways. These data together suggest that the chamazulene has diverse anticancer properties and shows significant potential as a treatment agent for breast cancer. Additional preclinical investigations are necessary to thoroughly clarify its mechanism of action and evaluate its potential in targeted therapeutic techniques.

## Data Availability

The original contributions presented in the study are included in the article/Supplementary Material, further inquiries can be directed to the corresponding author.
